# External validation of a clinical mathematical model estimating post-operative urine output following cardiac surgery in children

**DOI:** 10.1007/s00467-024-06456-9

**Published:** 2024-07-12

**Authors:** Orkun Baloglu, Bradley S. Marino, Samir Q. Latifi, Ayse Morca, Daniel S. Munther, Shawn D. Ryan

**Affiliations:** 1 Department of Heart, Vascular, and Thoracic, Division of Cardiology and Cardiovascular Medicine, Department of Integrated Hospital Care, Division of Critical Care, Children’s Institute, Cleveland Clinic Children’s, Cleveland Clinic Children’s Center for Artificial Intelligence (C4AI), Cleveland, OH USA; 2 Cleveland Clinic Lerner College of Medicine of Case Western Reserve University, 9500 Euclid Ave. M14, Cleveland, OH 44195 USA; 3 Department of Heart, Vascular, and Thoracic, Division of Cardiology and Cardiovascular Medicine, Cleveland Clinic Children’s, Cleveland, OH USA; 4 Department of Heart, Vascular, and Thoracic, Division of Cardiology and Cardiovascular Medicine, Department of Integrated Hospital Care, Division of Critical Care, Children’s Institute, Cleveland Clinic Children’s, Cleveland, OH USA; 5 Department of Heart, Vascular, and Thoracic, Children’s Institute, Cleveland Clinic Children’s, Cleveland Clinic Children’s Center for Artificial Intelligence (C4AI), Cleveland, OH USA; 6 Department of Mathematics and Statistics, Cleveland State University, Cleveland, OH USA

**Keywords:** Cardiopulmonary bypass, Congenital heart surgery, Mathematical modeling, Critical care, Pediatrics

## Abstract

**Background:**

This study aims to externally validate a clinical mathematical model designed to predict urine output (UOP) during the initial post-operative period in pediatric patients who underwent cardiac surgery with cardiopulmonary bypass (CPB).

**Methods:**

Children aged 0–18 years admitted to the pediatric cardiac intensive care unit at Cleveland Clinic Children’s from April 2018 to April 2023, who underwent cardiac surgery with CPB were included. Patients were excluded if they had pre-operative kidney failure requiring kidney replacement therapy (KRT), re-operation or extracorporeal membrane oxygenation or KRT requirement within the first 32 post-operative hours or had indwelling urinary catheter for fewer than the initial 32 post-operative hours, or had vasoactive-inotrope score of 0, or those with missing data in the electronic health records.

**Results:**

A total of 213 encounters were analyzed; median age (days): 172 (IQR 25–75th%: 51–1655), weight (kg): 6.1 (IQR 25–75th%: 3.8–15.5), median UOP ml/kg/hr in the first 32 post-operative hours: 2.59 (IQR 25–75th%: 1.93–3.26) and post-operative 30-day mortality: 1, (0.4%). The mathematical model achieved the following metrics in the entire dataset: mean absolute error (95th% Confidence Interval (CI)): 0.70 (0.67–0.73), median absolute error (95th% CI): 0.54 (0.52–0.56), mean squared error (95th% CI): 0.97 (0.89–1.05), root mean squared error (95th% CI): 0.99 (0.95–1.03) and R2 Score (95th% CI): 0.29 (0.24–0.34).

**Conclusions:**

This study provides encouraging external validation results of a mathematical model predicting post-operative UOP in pediatric cardiac surgery patients. Further multicenter studies must explore its broader applicability.

**Graphical abstract:**

**Figure:**
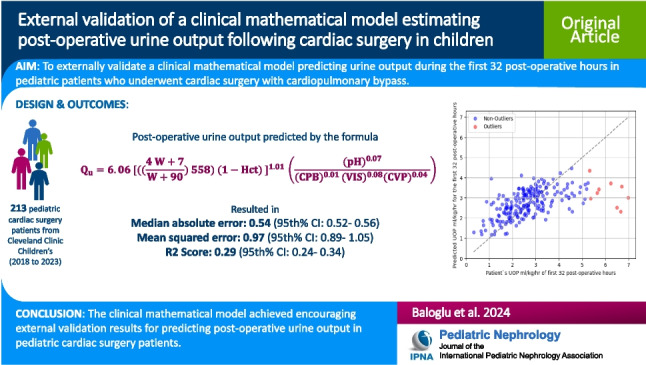
A higher resolution version of the Graphical abstract is available as Supplementary information

**Supplementary Information:**

The online version contains supplementary material available at 10.1007/s00467-024-06456-9.

## Introduction

Cardiopulmonary bypass (CPB) for cardiac surgery in children poses inherent challenges for post-operative care, given the physiological and anatomical intricacies of congenital heart diseases. Among the critical parameters that are highly associated with outcomes in the post-operative period is the patient's urine output (UOP), an indicator of kidney function and fluid balance. Decreased UOP is also used to diagnose low cardiac output syndrome (LCOS) in post-operative cardiac surgery patients [1–3]. Estimation of UOP during the initial post-operative phase could provide clinicians with useful information for tailored clinical management and LCOS prediction.

Recently, a mathematical model was developed utilizing a West Virginia University dataset from 2007 to 2013 to estimate UOP during the first 32 post-operative hours in children who underwent CPB for congenital heart surgery [4]. Due to the methodology of the West Virginia University dataset collection, 32 post-operative hours of time interval had to be chosen for the model’s output. The model variables were patient's weight, CPB duration, first hematocrit measurement, first arterial serum pH and first central venous pressure (CVP) measurement in pediatric cardiac intensive care unit (PCICU), and the initially calculated vasoactive-inotropic score (VIS) once the patient arrived in the PCICU. While the initial results from this model appeared promising, an external validation was required to determine its broader applicability. This study aimed to externally validate the mathematical model using a more recent dataset from Cleveland Clinic Children’s, with the objective to assess its viability as a clinical tool for predicting post-operative UOP in pediatric cardiac surgery patients.

## Methods

### Study design

This study was designed as a case series external validation study utilizing a dataset from Cleveland Clinic Children’s. This study was approved by the Institutional Review Board at The Cleveland Clinic and a waiver of consent was obtained (IRB# 23–725 titled as ‘Mathematical Modeling of Post-Operative Low Cardiac Output Syndrome approved on 7/19/2023) and the procedures were followed in accordance with the ethical standards of the responsible committee on human experimentation (institutional or regional) and with the Helsinki Declaration of 1975.

### Patients

Children admitted to Cleveland Clinic Children’s PCICU between 04/2018 and 04/2023 who were aged 0 to 18 years and underwent cardiac surgery with CPB were included. Patients were excluded if they had pre-operative kidney failure requiring kidney replacement therapy (KRT), re-operation or extracorporeal membrane oxygenation or KRT requirement within the first 32 post-operative hours or had indwelling urinary catheter for fewer than the initial 32 post-operative hours, or had VIS of 0, or those with missing model input data in the electronic health records (EHR).

### Data variables

Demographics, type of cardiac surgery, and model input and output variables were collected. The model input variables were the patient's weight at the time of the cardiac surgery, the duration of CPB in minutes, first measured arterial serum pH, first calculated VIS, and first measured CVP upon arrival to PCICU, defined as POD#0 variable. Since the purpose of the model was to predict post-operative UOP using variables available at the time of PCICU admission, and diuretics are not administered immediately upon PCICU arrival and other fluid losses such as chest tube output are unknown at the time of PCICU admission, those were not included as input variables for the model. The only output variable was the patient’s UOP in ml/kg/hr calculated for the first 32 post-operative hours. All data was manually extracted from the EHR.

### Statistical analysis

Descriptive analyses were performed for demographics, type of cardiac surgery, and model and output input variables. For patients who had more than one recorded cardiac surgery in the dataset, only the first encounter was included in analyses.

The mathematical model equation (Eq. 1) was used to predict a patient’s UOP in ml/kg/hr for the first 32 post-operative hours [4].

1 $${Q}_{u}=6.06 [(({\frac{4 W+7}{W+90}) 558)\;(1-Hct) ]}^{1.01 }\left(\frac{(p{H)}^{0.07}}{(CP{B)}^{0.01 }(VI{S)}^{0.08}(CV{P)}^{0.04}}\right)$$

where ‘*W’* is patient’s weight in kilograms at the time of cardiac surgery, *Hct* is the POD#0 hematocrit in decimals, ‘*pH’* is POD#0 arterial serum pH, ‘*CPB’* is duration of CPB in minutes, *‘VIS’* is the POD#0 VIS score, *‘CVP’* is POD#0 CVP in mmHg. The UOP in ml/kg/hr for the first 32 post-operative hours was calculated by dividing the ‘*Qu’* by the patient’s weight in kg and 32 (see Supplemental File 2 for the model built in an Excel file).

First, the model was tested in the entire dataset. Second, the model was tested and compared in patients < 12 months old vs. ≥ 12 months old and patients with STAT category < 3 vs. STAT category ≥ 3 as subgroup analyses. The model’s predictive performance was evaluated by mean absolute error (MAE), median absolute error (MedAE), mean squared error (MSE), root mean squared error (RMSE), and R2 score.

The following *Python* libraries were used in statistical analyses and graphing the results: *pandas: 1.4.4, numpy: 1.21.5, scipy: 1.9.1, sklearn: 1.0.2, matplotlib: 3.5.2.* The outliers for UOP were identified using the interquartile range (IQR) method, where values falling below IQR1 − 1.5 × IQR or above IQR3 + 1.5 × IQR were deemed outliers, with IQR1 and IQR3 representing the first and third interquartile quartiles, respectively. To estimate the 95% confidence intervals (CIs) of the model's predictive metrics, a subsampling strategy, taking 50% of the data in 10 separate iterations was used.

## Results

### Demographic and clinical data

A total of 213 encounters (see Supplemental File 1 for the details of excluded encounters) were analyzed; median age (days): 172 (IQR 25–75th%): (51–1655), weight (kg): 6.1 (IQR 25–75th%: 3.8–15.5), median UOP ml/kg/hr in the first 32 post-operative hours: 2.59 (IQR 25–75th%: 1.93–3.26) and post-operative 30-day mortality: 1 (0.4%). Further details on patient characteristics and model variables are summarized in Table 1.

**Table 1:** Summary of patient characteristics

Variable	
Total number of surgical encounters, *n*	213
Sex, male, *n* (%)	122 (57.2)
Age in days, median (IQR 25–75th%)	172 (51–1655)
Weight in kg, median (IQR 25–75th%)	6.1 (3.8–15.5)
STAT category, *n* (%)	
1	56 (26.2)
2	77 (36.1)
3	43 (20.1)
4	28 (13.1)
5	9 (4.2)
Cardiopulmonary bypass duration in minutes, median (IQR 25–75th%)	95 (74–134)
POD#0 Arterial serum pH, median, (IQR 25–75th%)	7.32 (7.28–7.36)
POD#0 Central Venous Pressure, median (IQR 25–75th%)	10 (7.0–13.0)
POD#0 VIS, median (IQR 25–75th%)	7.0 (5.0–10.0)
POD#0 Hematocrit %, median (IQR 25–75th%)	43 (38–48)
UOP of the first 32 post-operative hours in ml/kg/hr, median (IQR 25–75th%)	2.59 (1.93–3.26)
30-day post-operative mortality, *n* (%)	1 (0.4)

*STAT* The Society of Thoracic Surgeons-European Association for Cardio-Thoracic Surgery; *POD#0* Post-operative day 0; *VIS* Vasoactive-inotropic score; *UOP* Urine output

### The model’s predictive performance

**Fig. 1:**
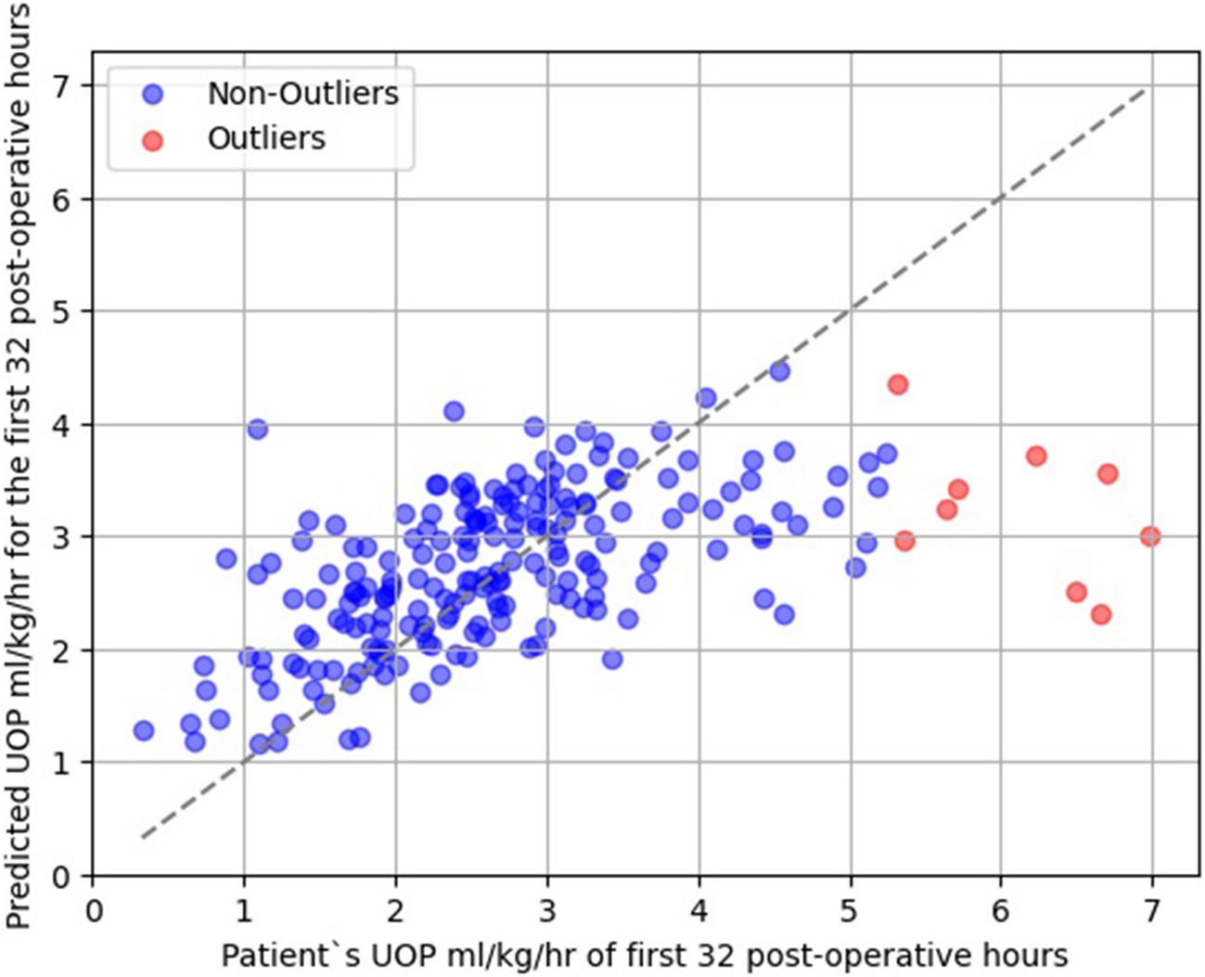
Comparison of predicted versus actual urine output (UOP) in pediatric cardiac surgery patients. The figure demonstrates a scatter plot illustrating the relationship between the UOP values predicted by the mathematical model (y-axis) and the actual observed UOP from all the patients (*N* = 213) (x-axis) during the initial 32 post-operative hours. Outliers, as defined by the interquartile range methodology described in the “Methods” section, are highlighted in red. The line of perfect agreement (45-degree line) is also depicted, serving as a reference for perfect prediction

The external validation of the model across all patients and different patient subgroups is summarized in Table 2. Overall, the model achieved MAE of 0.70 (95% CI: 0.67–0.73), MedAE of 0.54 (95% CI: 0.52–0.56), MSE of 0.97 (95% CI: 0.89–1.05), RMSE of 0.99 (95% CI: 0.95–1.03) and R2 score of 0.29 (95% CI: 0.24–0.34) in the entire dataset of 213 patients. Figure 1 demonstrates the predicted UOP values versus the actual observed UOP from all patients (*N* = 213) and highlights the outliers as defined in the “Methods” section.

**Table 2:** Summary of the model’s predictive performance in the entire dataset and the subgroups

Performance metric	All patients (*N* = 213)	Patients < 12 months (*N* = 128)	Patients ≥ 12 months (*N* = 85)	Patients with STAT category < 3 (*N* = 133)	Patients with STAT category ≥ 3 (*N* = 80)
Mean absolute error (95% CI)	0.70 (0.67–0.73)	0.84 (0.74–94)	0.58 (0.50–0.65)	0.72 (0.59–0.87)	0.75 (0.64–0.87)
Median absolute error (95% CI)	0.54 (0.52–0.56)	0.65 (0.58–0.69)	0.46 (0.35–0.53)	0.53 (0.40–0.65)	0.65 (0.54–0.81)
Mean squared error (95% CI)	0.97 (0.89–1.05)	1.26 (0.99–1.57)	0.80 (0.42–0.99)	1.11 (0.73–1.56)	0.91 (0.61–1.30)
Root mean squared error (95% CI)	0.99 (0.95–1.03)	1.12 (0.99–1.25)	0.89 (0.65–1.00)	1.05 (0.85–1.25)	0.95 (0.78–1.14)
R2 score (95% CI)	0.29 (0.24–0.34)	0.08 (0.01–0.14)	0.26 (0.18–0.37)	0.25 (0.16–0.42)	0.34 (0.19–0.47)

*CI* Confidence interval; *STAT* The Society of Thoracic Surgeons-European Association for Cardio-Thoracic

In patients younger than 12 months, the model showed MAE of 0.84 (95% CI: 0.74–94), MedAE of 0.65 (95% CI: 0.58–0.69), MSE of 1.26 (95% CI: 0.99–1.57), RMSE of 1.12 (95% CI: 0.99–1.25), and R2 score of 0.08 (95% CI: 0.01–0.14). In contrast, the model achieved better results in patients older than 12 months, with an MAE of 0.58 (95% CI: 0.50–0.65), MedAE of 0.46 (95% CI: 0.35–0.53), MSE of 0.80 (95% CI: 0.42–0.99), RMSE of 0.89 (95% CI: 0.65–1.00), and R2 score of 0.26 (95% CI: 0.18–0.37). In patients categorized with STAT category of < 3, the model resulted in MAE of 0.72 (95% CI: 0.59–0.87), MedAE of 0.53 (95% CI: 0.40–0.65), MSE of 1.11 (95% CI: 0.73–1.56), RMSE of 1.05 (95% CI: 0.85–1.25), and R2 score of 0.25 (95% CI: 0.16–0.42). Conversely, in patients with a STAT category ≥ 3 the model demonstrated slightly higher errors across metrics, with MAE of 0.75 (95% CI: 0.64–0.87), MedAE of 0.65 (95% CI: 0.54–0.81), MSE of 0.91 (95% CI: 0.61–1.30), RMSE of 0.95 (95% CI: 0.78–1.14), and R2 score of 0.34 (95% CI: 0.19–0.47).

## Discussion

In this external validation study of a recently developed mathematical model utilizing a dataset from Cleveland Clinic Children's, the model demonstrated a promising ability to predict post-operative UOP in pediatric cardiac surgery patients. The model could be a helpful tool for clinicians to gain early insights into the post-operative risk of oliguria by providing a quantifiable measure of expected urine output based on variables available at the time of PCICU admission. This foresight may allow for preemptive adjustments in fluid management and kidney support strategies during the post-operative period.

Comparison with existing literature is challenging since, to the best of our knowledge, this model stands as the only published tool predicting UOP for post-operative pediatric cardiac surgery patients [4]. Notably, the model showed enhanced predictive performance on the external dataset, achieving an MAE of 0.70 and RMSE of 0.99, compared to the MAE of 0.99 and RMSE of 1.4 reported in the original model development dataset [4]. This is particularly remarkable considering the validation dataset originated from a different institution, the Cleveland Clinic Children’s, as opposed to the original development dataset from West Virginia University and spans a different time interval (2018–2023) versus the original (2007–2013).

The significant reduction in the R2 score from 0.80 in the original study to 0.29 in the current external validation suggests that the model’s predictive power is considerably less outside of the development dataset. This decrease may reflect differences in patient populations, clinical settings, or disease severities not captured during model training. Moreover, the improved MAE in the external validation, despite a lower R2 score, highlights an important aspect of model evaluation. While R2 score measures the proportion of outcome variability explained by the model, MAE provides a straightforward average of error magnitudes. The observed improvement in MAE could indicate that the external dataset, despite its variability, may have lacked extreme values or outliers present in the original development dataset, thus skewing the average error lower. Unfortunately, the outliers were not reported in the original model development study which prevents us from comparing the differences in the outlier patients between the current study and the original report [4]. Additionally, similar trends are observed with RMSE, which also show improvements in the external validation. These metrics, MAE and RMSE, offer further insight into the model's error characteristics, suggesting that while the model may not explain a large proportion of variance, it consistently produces smaller prediction errors across these different measures. These findings also prompt a reevaluation of the model’s generalizability across diverse clinical environments. Moving forward, it is necessary to explore whether adjustments to the model or its inputs—potentially incorporating additional predictors or tuning parameters specifically for the external data—are necessary to enhance its robustness and applicability across varied settings.

Another significant finding is that all outliers as defined in the “Methods” section exhibited higher actual UOP than what the model predicted (Fig. 1). Such an error in prediction, higher actual UOP compared to the predicted UOP, may be considered more clinically acceptable than the alternative. It is also noted in Fig. 1 that there were more patients who had lower actual UOP than predicted UOP when the actual UOP was less than about 2.5 ml/kg/hr. Although, these patients were not classified as outliers by statistical criteria, it is important to mention that the model may result in clinically misleading predictions in some patients. Since UOP is a component of the LCOS definition the mathematical model that utilizes clinical variables collected immediately upon arrival to the PCICU might have the potential to serve as an adjunct to the LCOS prediction models [2]. Nevertheless, further multicenter research is required to validate this potential application.

The model showed worse performance in patients younger than 12 months compared to older patients. This difference in predictive performance might be largely attributed to the distinct physiological and clinical characteristics of infants. Unlike older children, infants experience more rapid physiological changes and exhibit greater variability in their response to treatment, which may not be sufficiently captured by the model parameters—these were potentially optimized for older age groups. Consequently, this misalignment leads to elevated prediction errors, as evidenced by the higher MAE, MSE, and RMSE in the younger subgroup. Additionally, the notably lower R2 score in infants suggests that the model fails to explain a significant portion of the variability in their data. This could be a result of the model not incorporating predictors that are specifically significant in infants or not appropriately weighting such variables. These findings underscore the necessity for developing tailored predictive models or adapting existing models to better cater to the unique characteristics of infants.

In patients with a STAT category < 3, which typically includes less complex and lower risk procedures, the model achieved an MAE of 0.72 (95% CI: 0.59–0.87) and an R2 score of 0.25 (95% CI: 0.16–0.42). This performance indicates a moderate level of predictive accuracy, suggesting that the model is reasonably effective in estimating outcomes for less severe cases. However, the confidence intervals are relatively wide, reflecting potential variability in the model’s performance, possibly due to a diverse range of less complex conditions within this subgroup. Conversely, for patients with a STAT category ≥ 3, representing more complex and higher-risk surgical interventions, the model showed a slightly higher MAE of 0.75 (95% CI: 0.64–0.87) and an improved R2 score of 0.34 (95% CI: 0.19–0.47). These results are particularly noteworthy as they suggest that the model, despite facing clinically complex scenarios, manages to capture a larger proportion of the variance in outcomes compared to less complex cases. Since the model’s performance was not reported in different age groups or STAT categories in the original study [4], it is not possible to compare the current subgroup analyses results with any other results in the literature.

The model and this study have limitations. One of the constraints is the model’s inability to provide predictions when the VIS is zero because of impossibility to calculate values when the denominator is zero (Eq. 1). Although, this limitation restricts the model’s application to clinical scenarios when the VIS is not zero, one may argue that prediction of UOP may not be as critical for patients with VIS of 0 at POD#0 since those could be considered as more hemodynamically stable and at lower risk of developing LCOS. The retrospective nature and single-center design are other notable limitations of the study.

Given the mentioned limitations of the current model and the results of this study, it is evident that the model can be further improved, particularly for infants, to enhance its predictive accuracy. Specifically, incorporating additional input variables could significantly reduce prediction errors and improve the R2 score. Subsequent multicenter studies are needed not only for testing the model's generalizability but also for identifying opportunities to refine the model.

## Conclusions

The study provided promising external validation results of the mathematical model predicting post-operative UOP in pediatric cardiac surgery patients. Further multicenter studies are needed to test the model’s generalizability and further improve its predictive performance.

## Supplementary Information

Below is the link to the electronic supplementary material.

Graphical abstract (PPTX 136 kb)

Supplementary file1 (DOCX 23 kb)

Supplementary file2 (XLSX 11 kb)

## Data Availability

Data sharing will be considered upon request.
