# Supplementary material for: External validation of a clinical mathematical model estimating post-operative urine output following cardiac surgery in children
Source: Pediatr Nephrol. 2024 Jul 12;39(11):3347–52. doi: 10.1007/s00467-024-06456-9 (PMC11413201; doi:10.1007/s00467-024-06456-9)
Supplement: Supplementary file 1 — Graphical abstract (PPTX 136 kb) [file 467_2024_6456_MOESM1_ESM.pptx]

## Slide 1
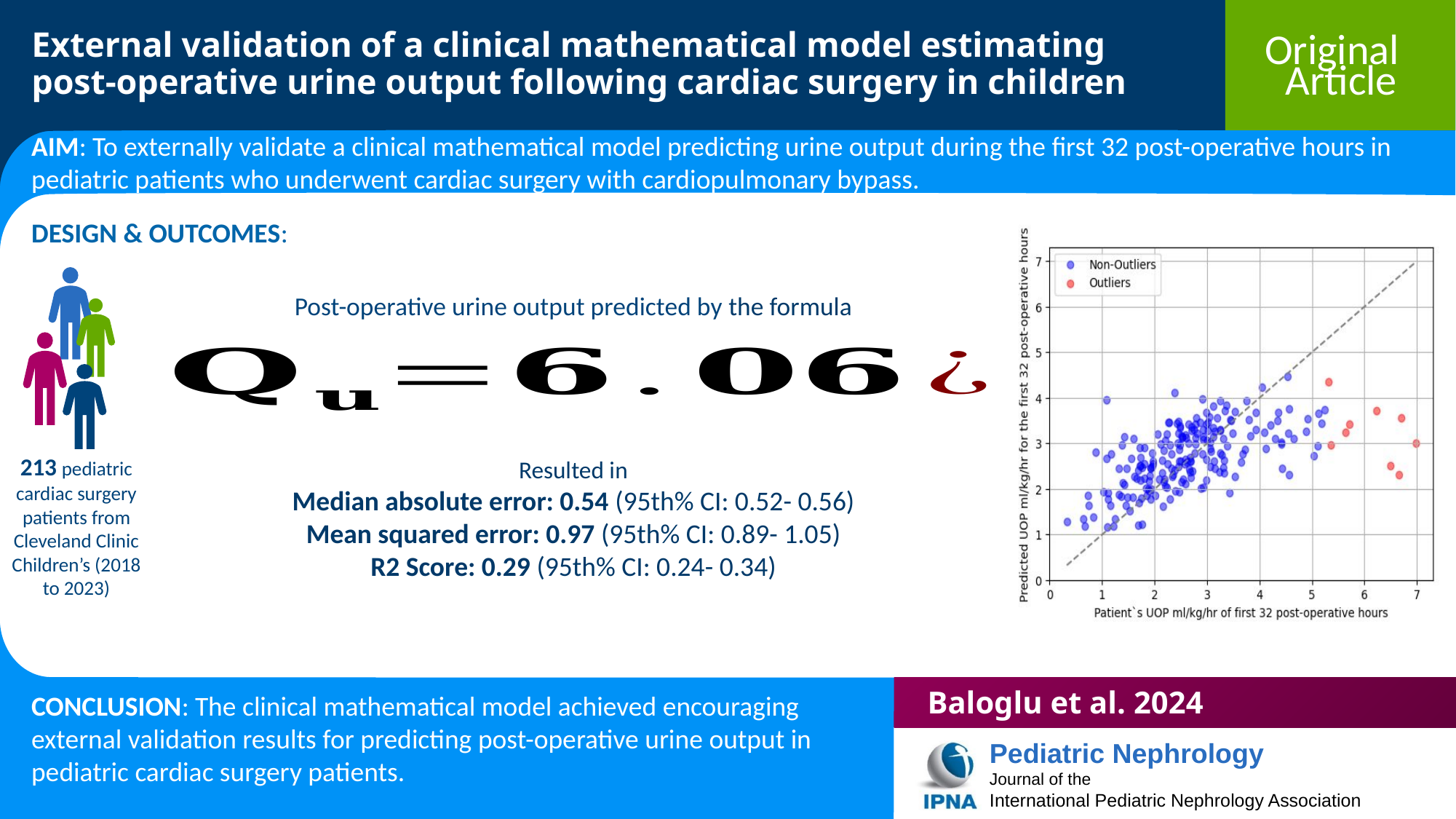

External validation of a clinical mathematical model estimating
post-operative urine output following cardiac surgery in children
AIM: To externally validate a clinical mathematical model predicting urine output during the first 32 post-operative hours in
pediatric patients who underwent cardiac surgery with cardiopulmonary bypass.
DESIGN & OUTCOMES:
Post-operative urine output predicted by the formula
213 pediatric cardiac surgery patients from Cleveland Clinic Children’s (2018 to 2023)
Resulted in
Median absolute error: 0.54 (95th% CI: 0.52- 0.56)
Mean squared error: 0.97 (95th% CI: 0.89- 1.05)
R2 Score: 0.29 (95th% CI: 0.24- 0.34)
Baloglu et al. 2024
CONCLUSION: The clinical mathematical model achieved encouraging external validation results for predicting post-operative urine output in pediatric cardiac surgery patients.
