# Supplementary material for: External validation of a clinical mathematical model estimating post-operative urine output following cardiac surgery in children
Source: Pediatr Nephrol. 2024 Jul 12;39(11):3347–52. doi: 10.1007/s00467-024-06456-9 (PMC11413201; doi:10.1007/s00467-024-06456-9)
Supplement: Supplementary file 2 — Supplementary file1 (DOCX 23 kb) [file 467_2024_6456_MOESM2_ESM.docx]

| **950 ENCOUNTERS** admitted Cleveland Clinic Children’s pediatric cardiac intensive care unit between April 2018 and April 2023, with patients aged 0 to 18 years. |
| --- |

| **567 ENCOUNTERS** |
| --- |

| **354 ENCOUNTERS EXCLUDED** for  3: Pre-operative kidney failure requiring kidney replacement therapy (KRT)  8: Re-operation <32 first post-operative hours  9: Extracorporeal membrane oxygenation <32 first post-operative hours  11: Requiring KRT <32 first post-operative hours  204: Indwelling urinary catheter in place <32 first post-operative hours  64: Vasoactive inotrope score =0  25: Missing data in the Electronic Health Records  30: Repeated encounters |
| --- |

| **213 ENCOUNTERS** included in the final analyses |
| --- |

| **383 ENCOUNTERS EXCLUDED** for  86: Pre-operative extracorporeal membrane oxygenation  247: No Cardiopulmonary bypass  50: Non cardiac surgeries |
| --- |
